# Reciprocal relationships between self-control and self-authenticity: a two-wave study

**DOI:** 10.3389/fpsyg.2023.1207230

**Published:** 2023-07-20

**Authors:** Qingqing Li, Xiaomei Ren, Zongkui Zhou, Jing Wang

**Affiliations:** ^1^Key Laboratory of Adolescent Cyberpsychology and Behavior (CCNU), Ministry of Education, Wuhan, China; ^2^Key Laboratory of Human Development and Mental Health of Hubei Province, School of Psychology, Wuhan, China; ^3^School of Psychology, Central China Normal University, Wuhan, China; ^4^Xixian High School Affiliated to Central China Normal University, Xinyang, China

**Keywords:** self-control, self-authenticity, reciprocal relationship, adolescent, longitudinal design

## Abstract

Both self-control and self-authenticity are critical to individuals’ mental health and social adjustment, but their relationship has received little attention. Research demonstrates that exerting self-control could help individuals achieve true self and might be promoted by perceiving authenticity. Accordingly, this study utilized a longitudinal design and investigated the temporal relationship between self-control and self-authenticity in a large sample of 2,982 Chinese adolescents (*M*_age_ = 17.53, *SD* = 0.84). Correlation analysis showed that participants possessing higher self-control were associated with greater self-authenticity. Cross-lagged path analysis revealed a reciprocal relationship between self-control and self-authenticity over time. Moreover, bivariate latent change score model indicated that self-control predicted an increase in self-authenticity across time, and vice versa. Overall, this study advances our understanding and suggests that restraining temptation and impulse can promote adolescents’ authenticity, and that the experience of authenticity, in turn, facilitates their self-regulation.

## 1. Introduction

Self-authenticity refers to the degree to which individual’s behavior is consistent with his/her values, beliefs, and cognitions (Kernis and Goldman, 2006; Wood et al., 2008). The importance of authenticity in psychological functioning and well-being has been recognized in psychological theories (e.g., self-determination theory, self-concordance model) and empirical studies (Sheldon and Elliot, 1999; Deci and Ryan, 2000; Wood et al., 2008; Schlegel et al., 2009; Sheldon, 2014). As the core component of self-regulation, self-control is defined as the ability to inhibit and alter dominant impulses to serve long-term and valued goals (Baumeister et al., 2007; De Ridder et al., 2012; Inzlicht et al., 2021). Converging evidence has indicated that high self-control is beneficial to individuals’ physical and psychological health, academic and work achievement, and social adjustment (Tangney et al., 2004; De Ridder et al., 2012; Allemand et al., 2019; Duckworth et al., 2019; Li et al., 2021).

Adolescence coincides with the transition from childhood to adulthood, when adolescent undergo rapid biological, social, and emotional development and change (Steinberg and Morris, 2001). The development of self-concept and self-control ability is a key task during the period of adolescence, which contributes to their performance in different fields of achievement and adaptation (e.g., academic, interpersonal). Notably, self-authenticity and self-control have gained lots of attention as the basic human strengths and virtues identified by positive psychology (Peterson and Seligman, 2004; Peterson et al., 2006). It is curious whether high self-control, i.e., restraining impulses in pursuit of long-term goals, leads to experience higher self-authenticity and, similarly, whether high self-authenticity promotes one’s self-control. Recently, emerging evidence has accumulated in exploring the link of self-control to self-authenticity, but some controversies and shortcomings remain. Therefore, exploring the relationship between self-control and self-authenticity has important practical implications in guiding adolescents in a period of self-identity confusion to bulid a positive self-concept and to promote the development of personality qualities.

### 1.1. Self-control predicts subjective authenticity

According to self-determination theory, pursuing goals that are aligned with individuals’ core interests and values could promote his/her authenticity (Ryan and Deci, 2000; Deci and Ryan, 2012). Self-control, as the ability to overcome impulses to serve valued goals, is assumed to bring higher self-authenticity. Researchers further reveal that individuals high on self-control are more inclined to adopt goal-directed and effective regulation strategies to reduce the experience of goal-irrelevant conflict (e.g., temptation, obstacle, Milyavskaya et al., 2021; Wenzel et al., 2021). When individuals engage in more natural and less constrained behaviors, they can express their “true” selves more easily and are less susceptible to external influence. Kokkoris et al. (2019) supported this proposition and suggested that resisting temptation could promote the experience of being aligned with one’s true self for individuals higher in rationalism. Moreover, based on cross-sectional survey, scenario-based experiments, and longitudinal designs, Ge and Hou (2021) found that self-control based on questionnaire measurement positively predicted self-authenticity, which in turn forecasted mental health. Interestingly, researcher recently found an actor-observer asymmetry that participants perceived impulsive behaviors as more authentic for others but self-regulated behaviors as more authentic for themselves (Garrison et al., 2022).

Some indirect evidence also suggests that resistance to temptation and persistence on long-term goals that are reflective of core needs and values are conductive to realizing one’ s potential and true self. For instance, Vainio and Daukantaitė, 2016 indicated that grit, a construct that overlaps with but differs from self-control, positively predicts various aspects of well-being through self-authenticity. Similarly, adolescents high on self-control ability are positively associated with promotion focus (Kim et al., 2019), positive affect (Lenton et al., 2013), and satisfaction of basic psychological needs (Milyavskaya et al., 2015a; Thomaes et al., 2017), which further contribute to their subjective authenticity. Nevertheless, there are studies showing that exerting self-control attenuates the tendency to view choice as indicative of preference and reduces subjective authenticity (Sela et al., 2017). English and John (2013) suggest that emotions are a prominent expression of one’s internal attributes, and emotional suppression may lead to the incongruence between inner-self and outer-behavior and result in subjective inauthenticity. Some researchers argue that individuals with high self-control often override spontaneous desires and adjust their behavior in favor of external constraints, social appropriateness, and long-term considerations, and thus are less likely to feel authentic (Sela et al., 2017).

### 1.2. Subjective authenticity predicts self-control

Existing literature has yet to directly investigated the predictive effect of self-authenticity on self-control. According to the organismic valuing theory, individuals have a natural growth motivation toward self-actualization and their “true self” (Rogers, 1964). The self-concordance model also posits that pursuing self-concordant goals (consistent with people’s developing interests and core values) would put sustained effort into achieving those goals (Sheldon and Elliot, 1999). Empirical research provides initial and indirect evidence that having a clear, confident, and consistent self-concept enables one to identify and prioritize self-initiated and valued goals, thus fostering effective self-regulation and goal perseverance in long-term goal pursuit (Higgins, 1996; Light, 2017; Schwartz et al., 2017; Wong and Vallacher, 2018). For instance, Wong and Vallacher (2018) used a daily diary design and found that fluctuations in the clarity of self-knowledge predicted fluctuations in goal perseverance. By contrast, people with low authenticity often act with a discrepancy between their inner experiences and outward behaviors, which leads to greater psychological conflict and lower functioning (Goldman and Kernis, 2002). Furthermore, Light et al. (2018) found that when participant received a reminder about attractive alternative goals, feeling uncertainty about oneself reduces one’s commitment to the focal goals. This suggests that people who have confusion about oneself are susceptible to external influence, which may hinder them from exerting control efforts.

In summary, the effect of self-control on self-authenticity remains controversial in that high self-control or exerting self-control does not necessarily lead individuals to experience high self-authenticity. Whereas preliminary research suggests that self-authenticity could facilitate self-control, direct research evidence is lacking. Meanwhile, most prior work testing the relationship between self-control and authenticity used either cross-sectional designs, or between-person regression analysis with longitudinal designs, ignoring individual differences and the nesting of repeated assessments. Exploring changes of self-control and self-authenticity, and their covariation may guide future design of family rearing, school education, and personalized prevention efforts. Therefore, tethering within-person data analytic approaches with longitudinal study designs is needed to understand the relationship between self-control and subsequent changes in self-authenticity, and vice versa. However, to our knowledge, such a bidirectional relationship between self-control and self-authenticity has not been directly examined.

To address this research gap, the present study utilized a two-wave longitudinal design with cross-lagged panel model and bivariate latent change score model to examine the relationship between self-control and self-authenticity. The cross-lagged model is able to examine the predictive role of one variable on the other in a prospective manner after controlling for their prior levels. And the bivariate latent change score model can test if one variable is associated with future within-person changes in another variable by the next time point after accounting for the autoregressive effects of each variable from their prior levels. Based on the reviewed evidence, the present study proposed that there might be a reciprocal relationship between self-control and self-authenticity. Specifically, higher self-control would predict greater self-authenticity at the next time-point and a within-person increase, and higher self-authenticity would forecast greater self-control and a within-person increase by the next time-point.

## 2. Methods

### 2.1. Participants and procedure

In this study, a cluster sampling method was used to recruit all senior and junior students within the academic system from several randomly selected high schools in Henan and Hubei Province located in the central regions of China. The first wave (T1: October 2020) collected questionnaire data from 3,000 middle school students (*M*_age_ = 17.26, *SD* = 0.85). Because some T1 respondents changed majors, cases were lost from T1 to T2 (April 2021), and 2,539 valid questionnaires (55.10% girls, 15 ~ 20 years old, *M*_age_ = 17.53, *SD* = 0.84) were collected at T2 (attrition rate = 15.37%). To examine whether these cases were missing randomly, we created a dummy indicator (0 = missing, 1 = complete) and conducted t tests. No significant differences were found between those remaining and those left: *t*_SC_ = 0.26, *p* > 0.05; *t*_authenticity_ = 0.86, *p* > 0.05. Information on the following demographic characteristics were collected: age, gender, family economic income (1 = below 1,000￥; 2 = 1,001 ~ 3,000￥; 3 = 3,001 ~ 5,000￥; 4 = 5,000 ~ 10,000￥; 5 = 10,001 ~ 20,000￥; 6 = above 20,000￥), residence (i.e., rural or urban area), and parent education (1 = primary school and below; 2 = middle school; 3 = high school degree and special school degree; 4 = undergraduate degree; 5 = graduate degree and above). All participants signed the informed consent document prior to the test and received an honorarium at the end of the study. Ethical approval of this study was granted by the Ethics Committee of the University.

### 2.2. Measurements

*Self-control* was measured using a brief and well-validated version of the Trait Self-Control Scale (Tangney et al., 2004), encompassing control over thoughts, emotional control, impulse control, performance regulation, and habit breaking. Participants were asked to answer 13 items on a 5-point Likert scale (e.g., “I am good at resisting temptation”) ranging from 1 (not at all like me) to 5 (very much like me) to indicate their general self-control. Items were averaged so that higher scores indicated greater self-control ability. In the current sample, Cronbach’s alpha of this scale in two waves were 0.83 (T1) and 0.84 (T2).

*Self-authenticity* was measured with the Authenticity Scale (Wood et al., 2008), which has been used to measure one’s true and authentic feeling and behavior, and contains 12 items. Three four-item subscales were used to assess three key aspects of self-authenticity: authentic living, which refers to individuals’ behavior and emotional expression that are consistent with their internal emotions, beliefs, and psychological states (e.g., “I live in accordance with my values and beliefs”); accepting external influence, which refers to the extent to which one accepts the influence of other people and the belief that one has to conform to the expectations of others (e.g., “I always feel I need to do what others expect me to do,” reversed); self-alienation, which refers to the degree of detachment between one’s conscious awareness and actual experience (e.g., “I feel out of touch with the real me,” reversed). Items were rated on a 7-point Likert scale (1 = does not describe me at all, 7 = describes me very well), with higher scores indicating a higher level of experienced authenticity. Previous research has supported the reliability and validity of the Chinese version of this inventory (Wang, 2015; Ge and Hou, 2021). In the present study, Cronbach’s alpha of this scale in two time points were both 0.84.

### 2.3. Data analysis

Following the descriptive analysis, measurement invariance model and cross-lagged panel model were conducted in M-plus 7.0, and bivariate latent change score model was conducted in R 4.1 software. A longitudinal confirmatory factor analysis (CFA) was used to compare the internal consistency of measurements over time, and simultaneously to assess the invariance model to determine whether a cross-lagged analysis was appropriate. A configural invariance model was first established to estimate all parameters across T1 and T2; then, a weak invariance model constrained factor loading to be equal across times was established; finally, a strong invariance model which included factor loads and intercepts to be equal across times. The changes in CFI values were evaluated to model differences. A ΔCFI <0.01 indicated a significant difference between the models (Cheung and Rensvold, 2002).

Then, we conducted a cross-lagged panel model analysis. In addition to calculating the cross-sectional correlations between the study variables, the analysis also include stability from T1 to T2 (i.e., auto-regressive path) and cross-lagged effects (i.e., reciprocal relationship between TSC and self-authenticity over time). The measurement model fit was evaluated based on the comparative fit index (CFI), Tucker-Lewis index (TLI), root–mean–square error of approximation (RMSEA), and standardized root–mean–square residual (SRMR). The following criteria were used to indicate the goodness of fit: CFI ≥ 0.90, TLI ≥ 0.90, RMSEA ≤0.08, and SRMR ≤0.08 (Hu and Bentler, 1999).

Although cross-lagged models can test reciprocal relationships, it cannot explicitly capture change patterns. Bivariate dual latent change score model is a highly flexible approach to examine the dynamic (i.e., time-lagged) reciprocal relationships related to individual differences in change (McArdle, 2009; Ferrer and McArdle, 2010). This approach can be utilized to test if self-control at a previous time-point would be related to change in self-authenticity by the next time-point (and vice versa). Specifically, factors representing individual informant reports are firstly created from observed item scores (i.e., both self-control and self-authenticity in two waves). Then, latent difference scores (i.e., Δ self-control, Δ self-authenticity) are created as second-order latent factors from the explicit factors representing individual informant reports. Conceptually, each change score (Δ) contains two major components that capture the change of the variable from the previous assessment and the effect of the other variable at the previous assessment. Pertinent to our research question are two key paths of this model that represent the estimated influence of an initial score of one variable on the subsequent changes in the other variable over time: (1) whether self-control changes (Δ self-control) from T1 to T2 are driven by self-authenticity at T1; (2) whether self-authenticity changes (Δ self-authenticity) from T1 to T2 are driven by self-control at T1.

## 3. Results

### 3.1. Common methods bias analysis

Because the data were collected from self-report questionnaires, common method deviation might occur. To check and test common method bias, Harman’s single-factor test using confirmatory factor analysis was conducted (Podsakoff et al., 2003). The present study conducted a factor analysis on all items of self-control and self-authenticity, and extracted a common factor from these items. The results showed that the interpretation rate of the first factor was 22.37%, less than 40%, indicating that there was no common method bias in the questionnaires used in this study.

### 3.2. Descriptive statistics and correlation analysis

Descriptive statistics for model variables were shown in Table 1. Skewness and Kurtosis values were within acceptable limits, suggesting normal distributions of these variables. As predicted, results showed that self-control was significantly and positively correlated with self-authenticity at two time points (*ps* < 0.01), suggesting that higher self-control was associated with greater self-authenticity.

**Table 1:** Descriptive statistics and correlations among variables (*N* = 2,982).

Variables	*M*	*SD*	skewness	kurtosis	1	2	3	4
Trait self-control (T1)	2.69	0.57	0.19	0.45	-			
Trait self-control (T2)	2.78	0.56	0.23	0.47	0.56**	-		
Self-authenticity (T1)	4.33	0.90	0.22	0.69	0.57**	0.39**	-	
Self-authenticity (T2)	4.31	0.81	0.57	0.84	0.37**	0.52**	0.56**	–

*N* = number; SD = standard deviation. **p < 0.01.

### 3.3. Measurement invariance

A longitudinal CFA was used to compare the measurements’ internal consistency over time by simultaneously assessing the invariance. As shown in Table 2, the CFI model changes in the study indicated that self-control had a strong equivalence and self-authenticity had a weak equivalence, suggesting that they did not change as a function of time.

**Table 2:** A longitudinal confirmatory factor analysis for measurement invariance.

	Model fit index					Model comparison		
	*χ^2^/df*	CFI	TLI	RMSEA	SRMR		Δ*χ^2^*	ΔCFI
**Self-control**								
Model 1	13.96	0.96	0.93	0.07	0.03			
Model 2	12.89	0.95	0.94	0.07	0.04	2 VS. 1	16.28**	0.003
Model 3	14.23	0.94	0.93	0.07	0.04	3 VS. 2	111.17***	< 0.001
**Self-authenticity**								
Model 1	22.05	0.97	0.90	0.09	0.05			
Model 2	18.46	0.96	0.92	0.08	0.06	2 VS. 1	19.60***	< 0.001
Model 3	14.93	0.96	0.93	0.07	0.06	3 VS. 2	0.19	0.91

Model 1 = the configural invariance model, Model 2 = the weak invariance model, Model 3 = the strong invariance model. **p < 0.01, ***p < 0.001.

### 3.4. Cross-lagged path model

**Figure 1:**
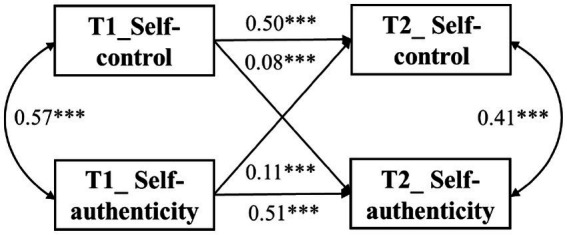
The cross-lagged panel models between self-control and self-authenticity. ***p < 0.001.

Four models were established separately to test the cross-lagged relationship between self-control and self-authenticity. Results showed that compared to Model 4 (baseline model), Model 5 (baseline model with paths from self-control to self-authenticity), and Model 6 (baseline model with paths from self-authenticity to self-control), Model 7 (baseline model with cross-lagged paths) improved model fit (see Table 3). Furthermore, cross-lagged model analysis (see Figure 1) showed a reciprocal relationship between self-control and self-authenticity, suggesting that the two variables positively predicted each other.

**Table 3:** Model comparisons of cross-lagged path analysis.

	Model fit index					Model comparison		
	χ^2^/df	CFI	TLI	RMSEA	SRMR		Δχ^2^	Δdf
Model 4	5.68	0.97	0.96	0.04	0.04			
Model 5	3.82	0.98	0.98	0.03	0.02	5 VS.4	23.27***	1
Model 6	3.08	0.99	0.98	0.03	0.02	6 VS.4	32.20***	1
Model 7	1.86	0.99	0.99	0.02	0.01	7 VS.4	46.75***	2
						7 VS.5	23.49***	1
						7 VS.6	14.45***	1

Model 4 = baseline model, Model 5 = baseline model with paths from self-control to self-authenticity, Model 6 = baseline model with paths from self-authenticity to self-control, Model 7 = baseline + bidirectional paths. ***p < 0.001.

### 3.5. Bivariate latent change score model

**Figure 2:**
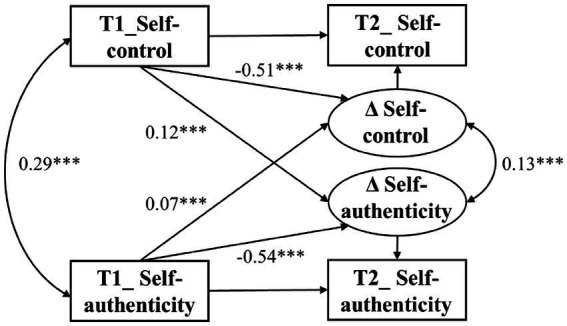
Bivariate latent change score models between self-control and self-authenticity. ***p < 0.001.

The bivariate latent change score model was established to examine the predictive effect of self-control on the within-person changes of self-authenticity, and vice versa. The latent change model fit the data adequately, *χ^2^*_(12)_ = 23.15, *p* < 0.05; CFI = 0.98, TLI = 0.99; RMSEA = 0.02, 90% [CI: 0.01, 0.03]; SRMR = 0.01. As portrayed in Figure 2, self-control positively predicted Δ self-authenticity (*β* = 0.12, *SE* = 0.03, z = 4.39, *p* < 0.001), and self-authenticity positively predicted Δ self-control (*β* = 0.07, *SE* = 0.01, z = 5.41, *p* < 0.001), suggesting that the two positively predicted a significant increase in each other. Moreover, results showed that Δ self-authenticity was positively associated with Δ self-control (*β* = 0.13, *SE* = 0.01, z = 19.09, *p* < 0.001) and indicated that the changes in both self-control and self-authenticity were positively correlated.

## 4. Discussion

The present study utilized a longitudinal design and investigated the associations of self-control and self-authenticity in a large sample of Chinese adolescents. Combining cross-lagged path analysis and bivariate latent change score model, our findings showed that there was a reciprocal relationship between self-control and self-authenticity over time. Specifically, greater self-control predicted higher levels of and an increase in self-authenticity across time; similarly, greater self-authenticity predicted higher levels of and an increase in self-control over time. This study advances our understanding and suggests that restraining temptation and impulse might promote adolescents’ authenticity, and that the experience of authenticity might facilitate their self-regulation.

Some authors argued that as people with high self-control are more restrained and self-disciplined in their daily lives, this group may have difficulty in achieving authenticity (English and John, 2013; Sela et al., 2017). In contrast to this view, the present study provide evidence that high levels of self-control are beneficial for adolescents to achieve a true self. Theoretically, our findings are in accordance with self-determination theory, which assumes that people have a natural propensity for growth and improvement (Ryan and Deci, 2000; Deci and Ryan, 2012). Moreover, Self-control contributes to the realization of this potential propensity by inhibiting and altering impulses to pursue long-term goals, which are aligned with their true self. Empirically, our findings are also consistent with prior studies showing that self-control facilitates self-authenticity (Kokkoris et al., 2019; Ge and Hou, 2021), suggesting that individuals’ decisions made under high self-control are more likely to be in line with their intrinsic true self. Taken together, adolescents with higher levels of self-control are more likely to control impulses and resist temptations in their lives, thus feeling more consistent and coherent with their true selves.

To our knowledge, few studies have directly explored the predicting effect of self-authenticity on self-control. Our results showing that higher self-authenticity forecasts increased self-control at the next time-point are in accordance with the organismic valuing theory and self-concordance model, which emphasize on the importance of self-concordant goals with long-term efforts. Some relevant empirical studies also support our current findings. For instance, Landa and English (2021) recently found that heightened authenticity was associated with lower emotion regulation efforts, while authenticity variability predicted greater effort to regulate emotions. Jiang et al. (2022) also suggests that when people feel confused about themselves, they will dismiss distal, larger valued goals and prioritize proximal, smaller satisfying goals. In addition, Milyavskaya et al. (2015a,b) found that want-to motivation improves self-regulation by reducing impulsive attraction to goal-disruptive temptations in the process of goal pursuit, suggesting that when individuals’ goal motivations are aligned with their own preferences, this authentic experience can promote self-regulatory ability. Therefore, feelings of true self-knowledge and true self-expression may be a prerequisite for achieving persistent self-regulation function in the pursuit of long-term goals.

Several limitations in the present study should be acknowledged. First, the participants were recruited from a population of Chinese high school students (i.e., two provinces located in the central regions), which may limit the generalizability of our findings. Future research should assess this finding in various samples across various ages and countries with different cultural backgrouds, as well as in samples with different orientations to happiness (e.g., hedonic vs. eudemonic). Second, measurement limitations included using self-report measures to assess trait-related constructs, which were vulnerable to subjectivity. Multiple measurements and designs with ecological validity and predictive effect, such as third-party reports (e.g., parents, teachers) and experience sampling (e.g., daily diary, ecological momentary assessment) should be considered in future research. For instance, daily diary, as a common form of experience sampling method, allows for the collection of participants’ experience, cognition, behavior across several successive natural days, providing the researcher with ecological data and evidence (Barrett and Barrett, 2001; Li et al., 2021). Third, although the current study confirms the reciprocal relationship between self-control and self-authenticity, we did not explore the explanatory mechanisms underlying this link, and future research could further explore this issue. While existing research focusing on adolescents’ self-control has received lots of attention, our understanding in their self-authenticity is lacking and future research should further examine the developmental status and trajectory of adolescents’ self-authenticity. Despite these shortcomings, the current findings have important implications for future research on promoting adolescents’ self-concept and self-regulation.

## 5. Conclusion

Research demonstrates that exerting self-control might help adolescents achieve true self and, in turn, perceiving authenticity might promote their development of self-control. The present study utilized a longitudinal design to investigate the associations of self-control and self-authenticity over time. Results indicated that a reciprocal relationship between self-control and self-authenticity and that self-control predicted an increase in self-authenticity across time, and vice versa. Overall, the present findings contribute to a deeper understanding of the longitudinal relationship between self-control and self-authenticity, and suggest that the development of self-concept and self-regulation is simultaneous and mutually reinforcing each other during adolescence.

## Data availability statement

The original contributions presented in the study are included in the article/supplementary material, further inquiries can be directed to the corresponding author.

## Ethics statement

The studies involving human participants were reviewed and approved by Central China Normal University, Ethic Committee. Written informed consent to participate in this study was provided by the participants’ legal guardian/next of kin.

## Author contributions

QL and JW: conceptualization. QL and JW: methodology and writing—original draft preparation. QL and XR: data curation. QL, XR, and JW: writing—review and editing. ZZ and JW: supervision. QL: project administration. All authors contributed to the article and approved the submitted version.

## Funding

This study was found by the Key Laboratory of Adolescent Cyberpsychology and Behavior (Central China Normal University), Ministry of Education (CCNUCYPSYLAB2022B08) and National Natural Science Foundation of Hubei Province.

## Conflict of interest

The authors declare that the research was conducted in the absence of any commercial or financial relationships that could be construed as a potential conflict of interest.

## Publisher’s note

All claims expressed in this article are solely those of the authors and do not necessarily represent those of their affiliated organizations, or those of the publisher, the editors and the reviewers. Any product that may be evaluated in this article, or claim that may be made by its manufacturer, is not guaranteed or endorsed by the publisher.
